# Canine and feline vector-borne diseases in Italy: current situation and perspectives

**DOI:** 10.1186/1756-3305-3-2

**Published:** 2010-01-11

**Authors:** Domenico Otranto, Filipe Dantas-Torres

**Affiliations:** 1 Dipartimento di Sanità Pubblica e Zootecnia, Facoltà di Medicina Veterinaria, Università degli Studi di Bari, 70010 Valenzano, Bari, Italy

## Abstract

In Italy, dogs and cats are at risk of becoming infected by different vector-borne pathogens, including protozoa, bacteria, and helminths. Ticks, fleas, phlebotomine sand flies, and mosquitoes are recognized vectors of pathogens affecting cats and dogs, some of which (e.g., *Anaplasma phagocytophilum*, *Borrelia burgdorferi*, *Dipylidium caninum*, *Leishmania infantum*, *Dirofilaria immitis*, and *Dirofilaria repens*) are of zoonotic concern. Recent studies have highlighted the potential of fleas as vectors of pathogens of zoonotic relevance (e.g., *Rickettsia felis*) in this country. While some arthropod vectors (e.g., ticks and fleas) are present in certain Italian regions throughout the year, others (e.g., phlebotomine sand flies) are most active during the summer season. Accordingly, control strategies, such as those relying on the systematic use of acaricides and insecticides, should be planned on the basis of the ecology of both vectors and pathogens in different geographical areas in order to improve their effectiveness in reducing the risk of infection by vector-borne pathogens. This article reviews the current situation and perspectives of canine and feline vector-borne diseases in Italy.

## Background

Canine and feline vector-borne diseases (VBDs) are caused by a wide range of pathogens, including viruses, bacteria, protozoa, and helminths, which are transmitted by a variety of vectors, such as ticks, fleas, mosquitoes, and phlebotomine sand flies. Some VBDs might be life-threatening in cats and dogs, they might develop after long incubation period making their diagnosis challenging, particularly because their clinical signs are not pathognomonic [1,2]. In addition, cats and dogs may eventually act as reservoirs of pathogens of zoonotic concern. A wide range of factors (e.g., climatic changes, human and animal population dynamics) may affect the occurrence and spread of VBDs [1]. However, the renewed interest on canine and feline VBDs of zoonotic concern is often not paralleled by publication of updated information on their distribution and ecology (e.g., seasonality and risk of exposure for susceptible hosts) in different geographical areas. This lack of knowledge, especially in cats, greatly impairs the development and implementation of effective preventive and control measures at national and regional levels.

In Italy, as well as in many other European countries, the aforementioned key information on parasitic arthropods and the pathogens they transmit is scant, anecdotic, and often outdated [3]. This is mainly due to the fact that most of the regional surveys or case reports on VBDs have been published in Italian journals or in proceedings of national scientific meetings, thus being in most of the cases not available to the international scientific community.

The present article reviews the current situation and perspectives of canine and feline VBDs in Italy. Additionally, the risk of transmission of vector-borne pathogens through different seasons is discussed in order to afford the implementation of effective control programs.

## Italy: geography and climate

**Figure 1:**
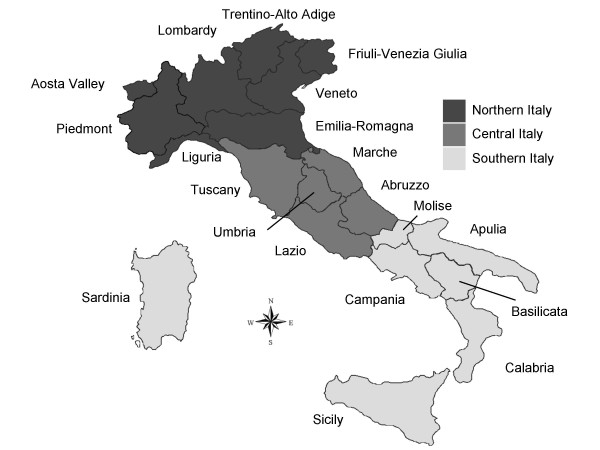
**Italy**. Three main areas with their respective administrative regions.

Italy is located in southern Europe and comprises the long, boot-shaped Italian Peninsula, the land between the peninsula and the Alps, and two main islands (Sicily and Sardinia). The country has a territory of 301,230 sq km, of which 294,020 sq km is land. It is geographically differentiated into three main areas which include administrative regions, namely northern (Liguria, Piedmont, Aosta Valley, Lombardy, Emilia-Romagna, Veneto, Trentino-Alto Adige, and Friuli-Venezia Giulia), central (Tuscany, Abruzzo, Umbria, Marche, and Lazio), and southern Italy (Campania, Apulia, Basilicata, Molise, and Calabria) including Sicily and Sardinia islands (Figure 1).

The climate of Italy is highly variable. Most of the inland northern areas have a continental humid climate whereas the coastal areas of the Liguria region and most of the peninsula have a typical Mediterranean temperate climate. The Italian peninsula has a large variety of habitats and environments, from the northern Alps through the Apennine Mountains to southern Italian and island regions. The geographical and climatic features, among other factors, contribute to the development of arthropod vectors, which may be present during most of the year, as is the case of ticks [4], fleas [5], and the Asian tiger mosquito *Aedes albopictus *[6].

## Free-roaming dogs and cats and risk of VBDs

Free-roaming (i.e., stray) dogs and cats are often present in urban and rural areas representing a public health concern in Italy [7]. Stray dogs have unrestricted movements and have been classified as neighbourhood (or community) and feral dogs on the basis of the level of dependence on human care [8]. It has been estimated that about one million stray cats and dogs live in Italy [7]. The high number of stray cats and dogs is probably due to the fact that their euthanasia is prohibited in Italy and animal abandoning is still a common practice. In fact, the current Italian legislation obligates public health authorities to maintain municipal shelters [7], where dogs are housed through their entire life, providing government protection and assistance for them.

Cats and dogs living in public shelters in Italy may be at high risk of acquiring vector-borne pathogens, mainly because they are often untreated against ectoparasites, thus, representing an easy feeding source for them. In addition, the general conditions of these animals (e.g., poor nutrition) may contribute to susceptibility to some VBDs. Likewise, when infected, free-roaming cats and dogs are often neither monitored nor treated against vector-borne pathogens.

Travelling of dogs and cats (e.g., for holidays) from VBD-endemic areas into Italy and *vice versa *poses a risk for the introduction and dissemination of exotic pathogens if competent vectors are present. In a recent study, it was found that 62% of dogs infected by *Babesia canis *had a history of travel to East European countries [9]. The above phenomenon highlights the importance of establishing effective surveillance systems to avoid the importation of infected cats and dogs into and from Italy.

## Arthropod vectors and related pathogens affecting dogs and cats in Italy

Dogs and cats living in Italy are at risk of becoming infected by different vector-borne pathogens, including protozoa, bacteria, and nematodes (Tables 1 and 2). Some vector-borne pathogens are widespread throughout the country and their occurrence in a given geographical area is affected by the presence of their competent arthropod vectors (i.e., ticks, fleas, lice, phlebotomine sand flies, mosquitoes, and secretophagous non-biting flies). Indeed, the likelihood of a dog or a cat becoming infected by a vector-borne pathogen in a given area is greatly influenced by vector population density as well as by the prevalence of the infection within the vector population. The closer is the contact between vectors and hosts, the higher will be the risk of infection. However, cases of VBDs have been diagnosed in cats and dogs in some areas where the presence of the proven vectors is unknown. This apparent absence of vectors in certain areas is likely to be the result of the limited number of studies conducted in these areas rather than the lack of the vectors themselves.

**Table 1:** Vector-borne pathogens affecting dogs in Italy.

Pathogen	Vector(s) ^a^	Geographical distribution ^b^
*Anaplasma phagocytophilum*	*Ixodes ricinus*, *Rhipicephalus sanguineus *(?)	C, Sardinia [16,82-84]
*Anaplasma platys*	*R*.* sanguineus *(?)	C, S [15,48,85,86]
*Babesia canis*	*Dermacentor reticulatus *(?), *R*.* sanguineus *(?)	C, N [14,54]
*Babesia vogeli*	*R*.* sanguineus*, *I*. *ricinus *(?)	C, S, N [54]
*Babesia gibsoni*	*R*.* sanguineus *(?)	S [52,87]
*Bartonella henselae*	*R*.* sanguineus *(?)	Sardinia [88]
*Bartonella vinsonii berkhoffii*	*R*. *sanguineus *(?)	S [17]
*Borrelia burgdorferi*	*I*.* ricinus*	C, N, S [20-22 ,24]
*Dirofilaria immitis*	*Anopheles maculipennis*, *Aedes albopictus*, *A*. *cinereus*, *A*.* geniculatus*, *A*.* detritus*, *A*.* punctor*, *Coquillettidia richiardii*, *Culex modestus*, *C*.* pipiens*, *C*.* torrentium*	C, N, S [33]
*Dirofilaria repens*	*A*.* albopictus*, *A*. *maculipennis*, *C*.* pipiens*	C, N, S [33]
*Acanthocheilonema grassii*	*R*.* sanguineus*	S [89]
*Acanthocheilonema dracunculoides*	*R*. *sanguineus*	S [90]
*Acanthocheilonema reconditum*	*R*.* sanguineus*	S [59]
*Dipylidium caninum*	*Ctenocephalides canis*, *C*. *felis*	C, N, S [91]
*Ehrlichia canis*	*R*. *sanguineus*	C, N, S, Sardinia [48-50]
*Hepatozoon canis*	*R*.* sanguineus*	C, N, S [14,92]
*Leishmania infantum*	*Phlebotomus ariasi*, *P*. *neglectus*, *P*.* perniciosus*, *P*.* perfiliewi*, *P*.* tobbi *(?)	C, N, S [33]
*Rickettsia conorii*	*R*. *sanguineus*	S [51,93]
*Thelazia callipaeda*	*Phortica variegata*	S, N [38,61]

Abbreviations: C, central Italy; N, northern Italy; S, southern Italy.

^a ^Question mark (?) indicates "suspected vector" to dogs.

^b ^Geographical distribution refers to the detection of pathogens either in dogs or in the vectors.

**Table 2:** Vector-borne pathogens affecting cats in Italy.

Pathogen	Vector(s) ^a^	Geographical distribution
*Anaplasma phagocytophilum*	*Ixodes ricinus *(?), *Rhipicephalus sanguineus *(?)	S [86]
*Bartonella henselae*	*Ctenocephalides felis *(?)	N [94,95]
*Bartonella clarridgeiae*	*C*. *felis *(?)	N [72]
*Dirofilaria immitis*	*Culex *spp. (?)	N [34,96]
*Dipylidium caninum*	*C*. *felis*	C, S [97]
*Acanthocheilonema grassii*	*R*. *sanguineus *(?)	C [98]
*Leishmania infantum*	*Phlebotomus perniciosus *(?), *Phlebotomus *spp. (?)	C [31,32]
*Rickettsia felis *^b^	*C*. *felis *(?)	N [26]
*Thelazia callipaeda*	*Phortica variegata *(?)	N, S [61]

Abbreviations: C, central Italy; N, northern Italy; S, southern Italy.

^a ^Question mark (?) indicates "suspected vector" to cats.

^b ^*Rickettsia felis *DNA has been detected in *C*. *felis *fleas, but no evidence of infection in cats has been shown [26]. The pathogenic role of *R*. *felis *in cats is yet to be demonstrated [99].

**Figure 2:**
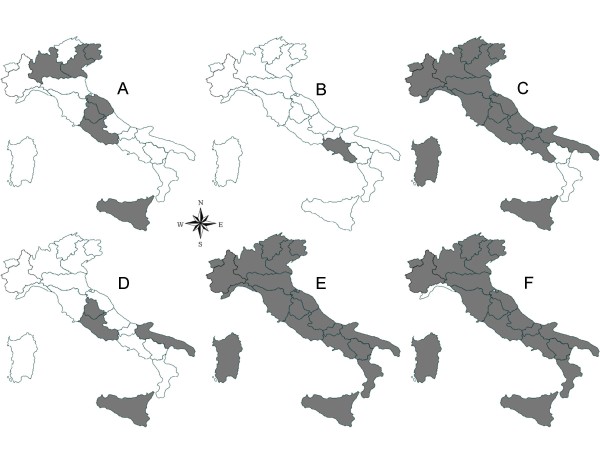
**Distribution of major vector-borne protozoa and of *Dirofilaria immitis *infecting dogs in Italy**. A, *Babesia canis*. B, *Babesia gibsoni*. C, *Babesia vogeli*. D, *Hepatoozon canis*. E, *Leishmania infantum*. F, *Dirofilaria immitis*.

**Figure 3:**
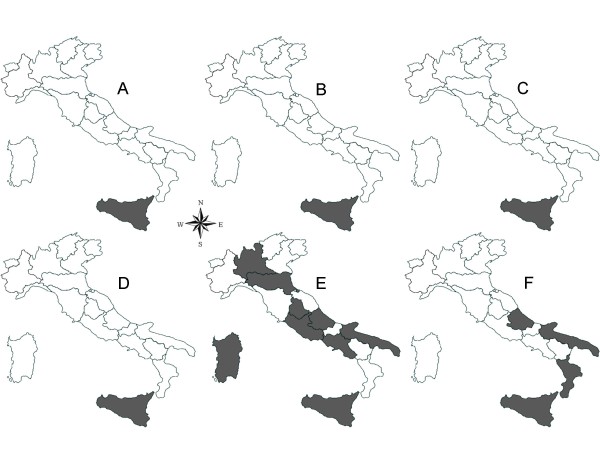
**Distribution of major vector-borne bacteria infecting dogs in Italy**. A, *Anaplasma phagocytophilum*. B, *Anaplasma platys*. C, *Borrelia burgdorferi*. D, *Coxiella burnetii*. E, *Ehrlichia canis*. F, *Rickettsia conorii*.

The geographical distribution of the main vector-borne pathogens affecting cats and dogs in Italy is reported in Figures 2 and 3. The maps have been elaborated based on data available in the literature, information provided by the Istituto Zooprofilattico della Sicilia (the reference centre for VBDs in Italy), and authors' unpublished data. Once again, the absence of certain pathogens in some regions (e.g., *Babesia vogeli *in Basilicata and Calabria regions) might be due the lack of studies carried out in these regions.

**Figure 4:**
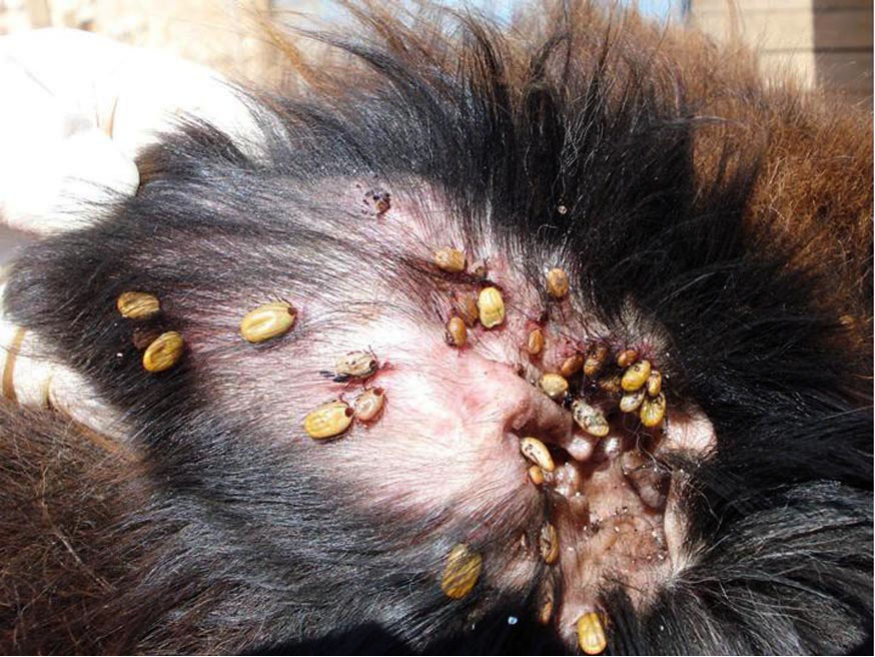
**Rhipicephalus sanguineus**. Several females attached to the ear of a dog from southern Italy.

*Rhipicephalus sanguineus *(the brown dog tick) is among the most important arthropod vectors involved in the transmission of pathogens affecting dogs worldwide [10]. In Italy, *R*.*sanguineus *(Figure 4) is the most common tick species infesting dogs [11-14]. It is the major vector of *B*.*vogeli*, *Ehrlichia canis*, *Hepatozoon canis*, and *Rickettsia conorii *and it is a putative vector of many other pathogens including *B*.*canis*, *Babesia microti*-like piroplasm (Spanish isolate), *Anaplasma platys *[14,15], and *Anaplasma phagocytophilum *[16]. Following the recent retrieval of *Bartonella vinsonii berkhoffii *genotypes II and III in dogs from southern Italy, a potentially new strain or species of *Bartonella *was detected in salivary glands of *R*.*sanguineus *ticks and in a dog [17]. Some of these pathogens (e.g., *E*.*canis*) are passed to the subsequent tick developmental stage (i.e., from larvae to nymphs and from nymphs to adults) transstadially [18]. Additionally, certain pathogens, such as *R*. *conorii*, might be maintained over several tick generations by transovarial transmission [19]. In this case, not only nymphs and adults but also larvae might play a role in transmitting the infection.

Other tick species (e.g., *Ixodes ricinus*, *Rhipicephalus turanicus*, *Hyalomma marginatum*, *Ixodes hexagonus*, *Rhipicephalus bursa*, *Dermacentor marginatus*, and *Dermacentor reticulatus*) have also been found on dogs in different Italian regions [4,14] and the potential role as vectors of some of them has been speculated upon. For instance, it has recently been suggested that *I*. *ricinus *could act as a vector of *B*. *vogeli *in central and northern Italy [14]. *Ixodes ricinus *is the major vector of *Borrelia burgdorferi *sensu lato and different "genospecies" have been detected in ticks collected in northern and central Italy [20-22]. Although an early experimental study failed to demonstrate the pathogenicity of *B*. *burgdorferi *(BITs1 Italian strain) in four beagles [23], anti-*B*. *burgdorferi *antibodies have been detected by indirect immunofluorescence antibody assay in a dog from Sicily presenting with fever, gait abnormalities, and diffuse pain [24]. Cats have also been found infested by ticks (e.g., *R*. *sanguineus*) in Italy [25] but the veterinary significance of this parasitism is largely unknown.

Fleas and lice are vectors of pathogens affecting dogs and cats worldwide [1], but little is known about their ecology and vectorial role in Italy. *Ctenocephalides felis *is the most common flea species infesting dogs and cats in Italy followed by *Ctenocephalides canis *[5,26]. Recent investigations have revealed the occurrence of *Rickettsia felis *DNA in *C*. *felis *collected from dogs and cats in different regions of Italy [26,27], pointing out the public health relevance of fleas infesting dogs and cats in this country. The prevalence of *R*. *felis *DNA in fleas was significantly higher in north-eastern (23.2% of 112 fleas) than in south-western (7.1% of 169) Italy [26]. Moreover, the positivity was higher among fleas collected from cats (17.6% of 74) than from dogs (10.2% of 246), although the number of fleas from cats tested was much lower when compared to those from dogs [26].

Lice (e.g., *Trichodectes canis*) are intermediate hosts of *Dipylidium caninum*, but there is no information about their role as vectors of pathogens infecting dogs or cats in Italy.

Phlebotomine sand flies (e.g., *Phlebotomus ariasi*, *Phlebotomus neglectus*, *Phlebotomus perniciosus*, and *Phlebotomus perfiliewi*) are vectors of *Leishmania infantum *[28], which is a pathogen of great zoonotic relevance affecting dogs [29] and occasionally cats [30-32] in Italy. By comparing recent entomological surveys with the historical data available, it has been recorded that there is an increase in terms of population density and geographical range of *P*. *perniciosus *and *P*. *neglectus *from southern and central to northern Italian areas [29]. The above phenomena might have ultimately contributed to the establishment of phlebotomine sand fly vectors of *L*. *infantum *into previously free areas in northern Italy and might represent an important issue to be considered while predicting the spread of phlebotomine sand flies northward through central European countries [33].

In Italy, about 16 species of mosquitoes (e.g., *A*. *albopictus *and *Culex pipiens*) have been regarded as proven or suspected vectors of the filarid nematodes *Dirofilaria immitis *and *Dirofilaria repens*, the causative agents of cardiopulmonary and subcutaneous dirofilariosis, respectively, in cats and dogs [33-35]. The most common filarial species parasitizing dogs in Italy are *D*. *immitis *and *D*. *repens *whereas *Acanthocheilonema reconditum*, *Acanthocheilonema grassii *and *Acanthocheilonema dracunculoides*, which infest subcutaneous tissue and/or muscular fasciae, are less diffused and of minor zoonotic concern [33].

*Phortica variegata *flies feed on lachrymal secretions around the eyes of a wide range of hosts, including humans and wild and domestic carnivores [36]. Males of this drosophilid fly have been shown to act as vectors of *Thelazia callipaeda *eyeworms under both experimental and field conditions [37,38].

From the information above, it becomes clear that dogs and cats living in Italy are exposed to the risk of infection by different vector-borne pathogens, some of which are of public health concern. The occurrence of some arthropod vectors throughout the year (e.g., ticks) and of others during summer months indicates that the risk of infection by vector-borne pathogens can vary in different areas of Italy according to the seasonal activity of their respective vectors.

## Seasonality of vectors in different Italian regions

The geographical distribution of VBDs is greatly influenced by the distribution and density of the different arthropod vectors which, in turn, is highly dependent on temperature and relative humidity of the different areas of Italy. For long time VBDs have been believed to be strictly linked to the seasonality in temperate areas and the role of climate and environmental factors on arthropod infection rate has been documented [39]. However, in the past decades, the 'seasonality paradigm' has been extensively debated and nowadays it cannot be considered more valid for many VBDs in the Mediterranean area [33]. In fact, there is evidence that microenvironment and climate changes have a significant impact on the distribution, transmission rates and prevalence of many VBDs. In the Mediterranean area, specifically in Italy, while some vectors (e.g., ticks, fleas, and some mosquito species) have been shown to be active throughout all the year [4-6], others (e.g., phlebotomine sand flies and *P*. *variegata*) display a typical seasonal activity [29,36]. This implies that dogs are at permanent risk of infection by certain tick-borne pathogens, such as *E*. *canis*. In the first case, a typical example is represented by *R*. *sanguineus*, the most widespread tick species in the world and probably the tick species transmitting the highest number of pathogens [10]. Due to its adaptability to human-modified ecosystems, this tick species is present through all the year in Italy [4]. Again, the presence of multiple tick species with overlapping seasonal patterns of activity in Italy (e.g., *I*. *ricinus *in northern regions, *D*. *marginatus *and *R*. *sanguineus *throughout the country) represents a further risk for tick-borne infections through the whole year [4].

As far as fleas are concerned, the occurrence of *C*. *felis *has been investigated in southern Italy. Although the prevalence of flea infestation was higher during the period between June and October, dogs were infested throughout the year [5] indicating a constant risk for flea-borne pathogen transmission. The results of this study are likely to be representative of other Mediterranean countries being also confirmed by surveys carried out in central Europe [e.g., [40]].

For a long time, mosquito vectors of *D*. *immitis *were thought to be more active during the summer months, mainly in humid climate areas of northern Italy and, at a lesser extent, in the remaining part of the Peninsula [41]. However, the introduction of *A*. *albopictus*, a known vector of *D*. *immitis *and *D*. *repens *[41,42], during the 1990s has been implicated in the appearance of new autochthonous foci of heartworm disease that have recently been reported in previously non-endemic areas of southern Italy (i.e., Apulia and Calabria regions) [33]. In addition, *A*. *albopictus *has adapted to the relatively low winter temperatures of Italy, rapidly increasing its populations through the country, developing many generations over the year in central and southern regions and overwintering as eggs in colder northern regions [6]. In contrast to other vectors of filarid nematodes, *A*. *albopictus *has a diurnal activity pattern [6]. This poses an additional threat to animals and humans, making them proportionally more exposed to *Dirofilaria *spp. in areas where both *A*. *albopictus *and other nocturnal mosquitoes (e.g., *C*. *pipiens*) occur in sympatry [41].

The occurrence and spread of canine leishmaniosis in a given area depends on several factors, including vector abundance and biting rates. In Italy, the activity of phlebotomine sand flies is seasonal and restricted to the summer months [43-47]. Indeed, recent entomological surveys carried out in northern [43], central [44] and southern regions [45] indicated that the activity of *P*. *perniciosus *(the most important vector of *L*. *infantum *in the Mediterranean area) is restricted to June and early October [29]. The activity of phlebotomine sand flies is predominantly nocturnal, but their biting activity pattern can vary according to species and foci [46,47].

The ecology and distribution of *P*. *variegata *has been studied in an area in southern Italy where *T*. *callipaeda *is highly endemic [36]. It has been shown that *P*. *variegata *has a seasonal pattern from May to October, being more active during July and August, at 20-25°C and 50-75% of relative humidity [36].

## Prevalence of infection by vector-borne pathogens in cats and dogs in Italy

It is difficult to assess the actual prevalence of infection by different vector-borne pathogens in cats and dogs in Italy due to the limited amount of data as well as to the difficulties in comparing information from studies using different diagnostic tools. Indeed, the prevalence of infection by vector-borne pathogens can also vary according to geographical region. For instance, the prevalence of *E*. *canis *infection in dogs estimated by serological surveys varied from 14.9% in southern Italy [48] to 46.7% in Sardinia [49]. Conversely, the overall prevalence of *E*. *canis *infection among 601 Italian dogs estimated by real-time PCR was lower than that recorded by serology, indicating that the infection is most prevalent in southern (9.7%) than in central (8%) and northern (2.9%) Italy [50]. Indeed, these discrepancies are due to the different methods used in each study, but also to the fact that the risk of *E*. *canis *infection varies among foci according to local factors (e.g., vector population density and activity patterns). *Anaplasma platys *infection has been molecularly detected in kennelled dogs in central (23%) [48] and southern Italy (11.3%) [15] as well as in dogs showing clinical signs of VDBs in southern Italy (4.3%) [51]. In addition, co-infection by *E*. *canis *and *A*. *platys *may also occur as recorded in a dog population from southern Italy where 44.4% of individuals infected by *A*. *platys *were co-infected with *E*. *canis *[48]. *Rhipicephalus sanguineus*, a proven vector of *E*. *canis*, has been suspected to act a vector of *A*. *platys *in Italy [14,15] and the high frequency of co-infection by *E*. *canis *and *A*. *platys *adds weight to this hypothesis. Similarly, high prevalence rates of anti-*R*. *conorii *antibodies (up to 74%) have been detected in dogs [51]. A study carried out in different Italian areas reported the occurrence of antibodies to *R*. *conorii *(56%) and *A*. *phagocytophilum *(3.7%) in dogs [51]. In a recent investigation carried out in southern Italy, 11.6% of 60 healthy dogs were positive for *Bartonella *spp. DNA [17]. Moreover, an uncultured *Bartonella *sp. (strain HMD) has been detected in five dogs, one of which was co-infected with *B*. *vinsonii berkhoffii *(genotypes II and III) [17]. These data indicate that dogs are exposed to multiple *Bartonella *species, some of which being of human health concern (i.e., *B*. *vinsonii berkhoffii*).

There are limited data on *Babesia *protozoa infecting dogs in Italy [51-54]. In a serological survey carried out in central and northern Italy it was recorded a mean prevalence of 34% of anti-*Babesia *antibodies in dogs with a decreasing trend from central to northern areas [14]. In the same survey, it has been recorded that 'kennel life style' and the age class 25-48 months represent risk factors for *Babesia *spp. infection [14]. The occurrence of *Babesia gibsoni *in Italy has recently been supported by molecular data [52]. Again, molecular investigations on *Babesia *spp. in blood samples from dogs with clinical signs compatible with VBDs have shown that *B*. *canis *is mainly detected in northern Italy (29.1%) whereas *B*. *vogeli *was detected mainly in central and southern Italy (16.3%) [54].

While canine dirofilariosis by *D*. *repens *has been considered for a long time to be mainly diffused in southern regions, *D*. *immitis *is endemic in northern regions with prevalence rates ranging from 22 to 80% [55,56] in dogs untreated with prophylactic drugs. In the past 20 years, *D*. *immitis *showed a relevant prevalence increase in endemic areas [56] and it was also recorded outside the main endemic area of the Po Valley, in provinces of north-eastern Italy previously regarded as non-endemic [57]. Furthermore, *D*. *immitis *has also become endemic in central regions such as Tuscany and Umbria [56,58]. A recent study carried out on dogs from southern Italy reported the occurrence of *A*. *reconditum *(16.5%) followed by *D*. *repens *(1.4%) and *D*. *immitis *(0.5%) in Campania [59]. The occurrence of *D*. *immitis *and *D*. *repens *has been also recorded in Apulia and Calabria with prevalence rates up to 1.6% [33]. The spread of *D*. *repens *in northern Italy [55] and the new foci of *D*. *immitis *recently detected in southern regions [33] indicate that dogs are at risk of both *Dirofilaria *spp. throughout the whole country. Until now, *D*. *immitis *infection in cats has been diagnosed mostly in northern Italy where the prevalence is about 18% in pet cats [60].

The highest prevalence of canine thelaziosis has been reported in some areas of southern Italy (Basilicata region), reaching up to 60% in certain municipalities [61]. In this area, *T*. *callipaeda *has also been found among different wildlife species, which have been implicated as its reservoirs [62].

For long time, stable endemic foci of canine leishmaniosis have been reported in southern and central Italy, with seroprevalence rates reaching up to 53.1% in some foci [63]. In a study conducted in the Apulia region (southern Italy), the yearly incidence rate of canine leishmaniosis among 168 dogs (92 farm and 76 kennel dogs) was calculated to be 9.5%, being higher (13.1%) among kennel dogs [64]. On the basis of recent data on phlebotomine sand fly collections and on human and canine leishmaniosis, new foci of canine leishmaniosis have been detected in northern regions where the disease was previously regarded as non-endemic [29]. As in the case of canine leishmaniosis, many other vector-borne pathogens infecting dogs and cats in Italy are not only important from a veterinary standpoint but might also represent a public health concern.

## Vector-borne pathogens of zoonotic concern affecting cats and dogs in Italy

Many vector-borne pathogens infecting cats and dogs in Italy may also be a threat to human health. These include many bacteria (e.g., *A*. *phagocytophilum*, *Bartonella henselae*, *B*. *vinsonii berkhoffii*, *B*. *burgdorferi*, *Coxiella burnetii*, *R*. *conorii*, and *R*. *felis*), nematodes (e.g., *D*. *immitis*, *D*. *repens*, and *T*. *callipaeda*), tapeworms (*D*. *caninum*), and *L*. *infantum*. In most of the cases, cats and dogs are unlikely to represent important reservoir hosts and their role in the transmission of these pathogens to humans is probably minor. On the other hand, cats and dogs may play a key role in the zoonotic cycle of transmission of some pathogens, such as *B*. *henselae *and *L*. *infantum*, respectively.

Dogs are primary hosts of vector-borne pathogens that may occasionally affect humans in Italy, including *D*. *immitis*, *D*. *repens *[65,66], and *T*. *callipaeda *[67]. Most importantly, dogs have been regarded as the main domestic reservoirs of *L*. *infantum *[29,68,69], which affects about 200 people annually in Italy [70]. Although *D*. *immitis *and *D*. *repens *have long been regarded as pathogens of veterinary concern, only recently these nematodes have been recognized as emerging zoonotic agents in Italy [65,66]. In particular, Italy is the first country in the world in the number of case reports (246 cases, from 1885 to 1999) of human dirofilariosis by *D*. *repens *[65] and dogs have been regarded as natural reservoirs of this filarid [71]. Similarly, the first cases of human thelaziosis in Europe have been diagnosed in the Piedmont region (northern Italy) in patients coming from north-western Italy and south-eastern France and now *T*. *callipaeda *has been recognized as an emerging parasite of humans in Italy as well as in other European countries [67]. Although dogs have also been found naturally infected by other pathogens (e.g., *A*. *phagocytophilum*, *C*. *burnetii*, and *R*. *conorii*) of public health concern in Italy [51], their role in the zoonotic transmission of these pathogens in this country is yet to be determined.

Cats have been found infected by emerging human pathogens, such as *B*. *henselae *and *Bartonella clarridgeiae *in Italy [72], and might be involved in the zoonotic transmission of these pathogens to man. The role of cats as additional domestic reservoirs of *L*. *infantum *has long been discussed [73] and a recent study has demonstrated that they can act as a source of *L*. *infantum *infection to *P*. *perniciosus *under laboratory conditions [32]. Recent studies have revealed high infection rates by *R*. *felis *(the causative agent of flea-borne spotted fever in humans) in fleas (*C*. *felis *and *C*. *canis*) collected from cats [26,27]. However, the role of cats in the epidemiology of flea-borne spotted fever is still to be determined [27].

Considering the zoonotic relevance of many vector-borne pathogens infecting cats and dogs in Italy, the control of VBDs they cause should not be only of veterinary concern but also a public health priority.

## Control and prevention of VBDs affecting cats and dogs in Italy

The control of VBDs requires a holistic approach, considering the distribution, and the ecology of the vectors and of pathogens they may transmit and the infection progression in infected animals. The current strategies for the control of arthropod vectors in dogs and cats have recently been reviewed elsewhere [2,74]. The control of ectoparasites on dogs and cats is largely based on the use of acaricides and/or insecticides (e.g., amitraz, fipronil, and permethrins) that are available in a number of formulations (e.g., pour-on, spot-on, baths, and insecticide-impregnated collars). Most of the commercially available ectoparasiticides for use in dogs and cats have a long-lasting effect, being safe for pets, their owners, and the environment. Moreover, they can present killing and/or anti-feeding effects, which may prevent pets from being bitten by arthropods for long periods, and thus from becoming infected by vector-borne pathogens [74]. The use of insecticides in combination with insect growth regulators (e.g., chitin synthesis inhibitors and juvenile hormone analogues) increases their ovicidal and/or larvicidal activity. When applied on dogs and cats, insect growth regulators also act in their sleeping areas which may be highly infested by fleas [74]. Indeed, the control of arthropod vectors such as fleas and ticks should be performed by using an integrated approach focusing on animals, but also on the environment.

The prevention of VBDs can be achieved by means of systematic application of acaricides and/or insecticides, prophylactic administration of drugs, and vaccination. However, as discussed previously in this review, the occurrence and the risk of acquiring VBDs in Italy can vary according to the geographical and seasonal distribution of their respective vectors. Thus, the success of any prevention strategy depends on the appropriate use of commercially available tools, which should not only consider the manufacturer's instructions but also the available information on the vector ecology in a given area or region.

In Italy, the chemoprophylatic use of ivermectin against third- and fourth-stage larvae of *D*. *immitis *and *D*. *repens *has long been recommended for dogs in highly endemic areas of northern regions [75]. In southern Italy, the same approach should be recommended only for dogs travelling to highly endemic areas. Ectoparasiticides with different modes of action and targeting different vector developmental stages have the potential to protect dogs and cats against infection by different vector-borne pathogens. For instance, field studies have shown the efficacy of different commercially available products in reducing the infection rate by *E*. *canis *[48] and *L*. *infantum *in dogs [45,76]. In Italy, vaccines against canine babesiosis are commercially available and should be recommended for dogs living in or travelling to endemic areas. Vaccines are also available against Lyme disease caused by *B*. *burgdorferi *sensu stricto, but not against the other species. The decision to vaccinate against Lyme borreliosis should be made based on a risk assessment of the individual dog that includes information about where the dog lives and how often it frequents a tick-infested area. As far as cats, no studies are available in the literature about the prevention of VBDs.

## Final considerations and perspectives

Undoubtedly, the general picture of the VBDs affecting dogs and cats in Italy is complex mainly due to the limited amount of published data on ecology and distribution of arthropod vectors and the pathogens they transmit in northern, central and southern areas of the country. Gaps in our knowledge are even worse for vector-borne pathogens infecting cats; for some of which (e.g., *R*. *felis*) almost no information is available.

Among arthropods parasitizing dogs and cats in Italy, ticks are the most important vectors of pathogens. Indeed, ticks are proven vectors of a large number of pathogens, present in a wide geographical distribution and, most importantly, they are active during the whole year. In particular, due to its high degree of adaptability to different microenvironments and its capability to occasionally feed on hosts other than dogs, *R*. *sanguineus *represents one of the major threats not only to dogs, but also to cats and humans [4,11]. Furthermore, the role of this tick as a putative vector of many species of other pathogens is a currently debated issue. For instance, the transmission of *Leishmania *parasites by *R*. *sanguineus *or by *C*. *felis *has received a renewed attention from the scientific community in recent years [10,77]. For instance, there is experimental evidence indicating that ticks could act as mechanical vectors of *L*. *infantum *via their ingestion [78] and a recent study has reported the detection of *L*. *infantum *kinetoplast DNA in salivary glands of *R*. *sanguineus *ticks collected from a dog living in a rural area in southern Italy [79]. Further studies are needed to assess the competence of ticks as vectors of *Leishmania *parasites from dog to dog which ultimately could open new perspectives for the control of this infection in areas where canine leishmaniosis is endemic but the primary vectors have not been found. Again, the retrieval of new strains or species of pathogens (e.g., *Bartonella *sp. strain HMD) [17] in salivary glands of *R*. *sanguineus *in southern Italysuggests that unrecognized vector-borne pathogens may exist, which could complicate the diagnosis and management of other endemic VBDs in dogs and cats. Furthermore, considering the importance of *R*. *sanguineus *as vectors of *R*. *conorii *and the medical relevance of *R*. *conorii *infection in Italy [80,81], further research on the role of dogs in the epidemiology of Mediterranean spotted fever should be carried out.

As a priority, it is necessary to establish effective national surveillance systems based on a regional level that, using standardized diagnostic procedures and protocols, could provide a clear picture on vector-borne pathogens circulating among dogs and cats (both pet and free-roaming ones) in different Italian regions. Data on the distribution of arthropod vectors (e.g., regional maps) and the pathogens they transmit would not only allow the assessment of the risk of the introduction of exotic pathogens into non-endemic areas but, more practically, could provide information to veterinarians and pet owners on timing and protocols that should be chosen for preventing VBDs in different Italian regions. In this regard, it is crucial to translate that data from research into changed practices by veterinarians and to ensure owners' compliance.

An affordable and reliable control of canine and feline VBDs should also be part of a strategy for the management of zoonotic infections in order to reduce the risk of pathogen circulation between pet animals and humans. However, these actions cannot be disconnected from political initiatives to reduce the number of free-roaming dogs and cats in Italy and to improve vector control programs in public kennels. Finally, it is crucial to implement a permanent surveillance system to prevent the entry and exit of exotic vector-borne pathogens through dogs and cats travelling with their owners to and from Italy.

## Competing interests

The authors declare that they have no competing interests.

## Authors' contributions

DO and FD-T contributed equally to conceiving and writing this article.
